# Current lipid extraction methods are significantly enhanced adding a water treatment step in *Chlorella protothecoides*

**DOI:** 10.1186/s12934-017-0633-9

**Published:** 2017-02-11

**Authors:** Xiaojie Ren, Xinhe Zhao, François Turcotte, Jean-Sébastien Deschênes, Réjean Tremblay, Mario Jolicoeur

**Affiliations:** 1 Research Laboratory in Applied Metabolic Engineering, Department of Chemical Engineering, École Polytechnique de Montreal, P.O. Box 6079, Centre-ville Station, Montreal, QC H3C 3A7 Canada; 2 Université du Québec à Rimouski, 310 allée des Ursulines, Rimouski, QC G5L 3A1 Canada

**Keywords:** *Chlorella protothecoides*, Lipid extraction, Water treatment, Two-stage solvent extractions, High extraction yield, High TAG ratio

## Abstract

**Background:**

Microalgae have the potential to rapidly accumulate lipids of high interest for the food, cosmetics, pharmaceutical and energy (e.g. biodiesel) industries. However, current lipid extraction methods show efficiency limitation and until now, extraction protocols have not been fully optimized for specific lipid compounds. The present study thus presents a novel lipid extraction method, consisting in the addition of a water treatment of biomass between the two-stage solvent extraction steps of current extraction methods. The resulting modified method not only enhances lipid extraction efficiency, but also yields a higher triacylglycerols (TAG) ratio, which is highly desirable for biodiesel production.

**Results:**

Modification of four existing methods using acetone, chloroform/methanol (Chl/Met), chloroform/methanol/H_2_O (Chl/Met/H_2_O) and dichloromethane/methanol (Dic/Met) showed respective lipid extraction yield enhancement of 72.3, 35.8, 60.3 and 60.9%. The modified acetone method resulted in the highest extraction yield, with 68.9 ± 0.2% DW total lipids. Extraction of TAG was particularly improved with the water treatment, especially for the Chl/Met/H_2_O and Dic/Met methods. The acetone method with the water treatment led to the highest extraction level of TAG with 73.7 ± 7.3 µg/mg DW, which is 130.8 ± 10.6% higher than the maximum value obtained for the four classical methods (31.9 ± 4.6 µg/mg DW). Interestingly, the water treatment preferentially improved the extraction of intracellular fractions, i.e. TAG, sterols, and free fatty acids, compared to the lipid fractions of the cell membranes, which are constituted of phospholipids (PL), acetone mobile polar lipids and hydrocarbons. Finally, from the 32 fatty acids analyzed for both neutral lipids (NL) and polar lipids (PL) fractions, it is clear that the water treatment greatly improves NL-to-PL ratio for the four standard methods assessed.

**Conclusion:**

Water treatment of biomass after the first solvent extraction step helps the subsequent release of intracellular lipids in the second extraction step, thus improving the global lipids extraction yield. In addition, the water treatment positively modifies the intracellular lipid class ratios of the final extract, in which TAG ratio is significantly increased without changes in the fatty acids composition. The novel method thus provides an efficient way to improve lipid extraction yield of existing methods, as well as selectively favoring TAG, a lipid of the upmost interest for biodiesel production.

**Electronic supplementary material:**

The online version of this article (doi:10.1186/s12934-017-0633-9) contains supplementary material, which is available to authorized users.

## Background

Microalgae is an attractive platform for lipid production [1, 2]. Microalgae cells can accumulate lipids at up to 20–50% of their cell dry weight [3], and which can be used as precursors for biodiesel production after a transesterification step [4, 5]. Algal lipids include polar lipids, which are normally structural such as phospholipids and glycolipids, and neutral lipids, which are mainly storage lipids such as mono-, di-, tri-acylglycerides (TAG) and sterols (ST) [6, 7]. TAGs represent the most preferable lipid class for biodiesel production since they contain fatty acids that can be removed from their glycerol frame, and transformed through transesterification reaction into fatty acid methyl esters (FAMEs) [8]. Significant efforts have been devoted to identify the genes and signals that regulate microalgae metabolism [9–12], and to optimize the upstream processing steps to generate lipid-rich cellular biomasses [13–23]. However, although the downstream process normally accounts for the major part of a bioprocess costs, only limited attention has been placed on the amelioration of lipid extraction protocols [3, 24, 25]; a step still considered as one of the major bottlenecks for commercial-scale biodiesel production [26]. Significant amounts of lipids are trapped in the cytoplasm by the cell walls and membranes, so lipid extraction efficiency thus greatly depends on cell disruption technique as well as on the polarity of the solvents used to remove lipids from the cell water phase [27–30]. For instance, some protocols favor imposing a high mechanical stress such as ultrasound treatment [3], resulting in a high cell disruption efficiency level. For comparison, a low shear stress approach such as using a hydrocyclone only leads to ~10% cell lipids extraction efficiency but microalgae cells remain viable [31]. Overall, the solvents perform lipid extraction, which explains the amount of work dedicated to identify the most efficient solvents combination.

A short series of solvent-based methods have been largely used to perform lipid extraction from various biological materials. The Folch method [32] consists in using chloroform–methanol (Chl/Met), and then the extracted solvent (chloroform) is washed with water to remove non-lipid substances. Bligh and Dyer then proposed a method based on Folch’s combining chloroform, methanol and water (Chl/Met/H_2_O), for lipid extraction from a wide range of biological materials [33]. More recently, because of concerns on biosafety, a less hazardous solvent mixture of dichloromethane/methanol (Dic/Met) has been proposed by Cequier et al. [34] as a substitute for Bligh and Dyer method. In addition, Drochioiu proposed a fast lipid assay with acetone extraction and turbidimetric reaction with sulfosalicylic acid, which requires only few milligrams of dry samples compared to grams for the above-mentioned methods, which limits their application to pilot and large scale production facilities [35]. These methods can be considered as references, or classical, in the field.

Comparative studies have been done with different microalgae species using different extraction systems. For the microalga *Chlorella vulgaris*, Araujo et al. [3] revealed that using Bligh and Dyer’s method (Chl/Met/H_2_O) [11, 12] is more efficient than Folch’s method (Chl/Met) [10], followed by Chen’s method using methanol/dichloromethane (Met/Dic) [36], while low efficiency levels were obtained for isopropanol/hexane [37] and soxhlet extraction using acetone [38]. Ryckebosch et al. [39] explored seven solvent mixtures at different ratios on *C. vulgaris*, and showed that extraction efficiency level was higher using chloroform/methanol 1:1, then for chloroform/methanol 2:1, followed by dichloromethane/ethanol 1:1, hexane/isopropanol 3:2, acetone, diethyl ether, and methyl-tert-butyl ether/methanol 10:3. For the marine microalgae *Tetraselmis* sp., Li et al. [24] revealed that Dic/Met [34] was the most efficient method, followed by propan/hexane (Pro/Hex) [40], Chl/Met/H_2_O [11, 12], supercritical CO_2_ [41] and finally ethanol/KOH [29]. For *Isochrysis galbana*, Grima et al. [42] have also compared seven solvent mixtures and found that the extraction efficiency level was higher for chloroform/methanol/H_2_O 1:2:0.8, followed by hexane/ethanol 1:2.5, hexane/ethanol 1:0.9, butanol, ethanol, ethanol/H_2_O 1:1, and hexane/isopropanol 1:1.5. As it can be seen, lipid extraction efficiency differs with biomass type as well as with the solvent mixture.

In this work, we thus test the hypothesis that a water treatment step added to current extraction protocols, between the two organic solvent extraction steps, increases cell material disruptions with an enhancement of lipid release from the cell. The four different extraction methods largely used for algal lipid extraction (Folch method with Chl/Met [32]; Bligh and Dyer method with Chl/Met/H_2_O [3, 33]; Cequier method with Dic/Met [34] and Drochioiu method with acetone [35]) were thus implemented with a water treatment. Results showed a significant improvement of the global lipid extraction efficiency, and especially for TAG, a precursor of biodiesel synthesis.

## Methods

### Experimental microalgae

*Chlorella protothecoides* was cultivated under heterotrophic condition for biomass and lipid accumulation [43]. The modified basal medium (MBM) [44] was used to maintain the inocula and to perform the experiments. Cells were collected at the exponential phase by centrifugation at 4000*g* for 10 min, and were vacuumed (remove extra water) and freeze-dried (VirTis, Advantage Plus EL-85) to determine the dry weight. Then the freeze-dried biomass was ground into a fine powder for subsequent extractions.

### Current lipid extraction methods

A mass of 35 mg of dried microalgae was used in each experiment. The four non-modified original extraction methods were applied in four control groups as detailed below.

### Method A: acetone [35]

35 mg of dry samples were extracted with 5 mL of acetone under ultrasound in ice water for 30 min, and centrifuged at 4000*g* at 4 °C for 5 min. Supernatants were transferred to a new test tube for lipid analysis, and the remaining cell pellets were re-extracted repeating the procedure.

### Method B: Chl/Met [32]

35 mg of dry microalgae samples were extracted with 7.5 mL of a mixture chloroform/methanol (2:1, v/v) under ultrasound in ice water for 30 min. The mixture was centrifuged at 4000*g* at 4 °C for 5 min. Cell pellets were kept for a re-extraction step and supernatants were transferred to a new test tube with 1.875 mL of H_2_O and shaken vigorously following a centrifugation at 4000*g* at 4 °C for 5 min. Then the lower layer of 5 mL chloroform with extracted lipids were pipetted out for lipid analysis. The remaining cell pellets were re-extracted repeating the procedure.

### Method C: Chl/Met/H_2_O [3, 33]

35 mg of dry microalgae samples were mixed and homogenized with 5 mL of methanol, 2.5 mL of chloroform and 5 mL of water. The mixture was treated under ultrasound in ice water for 20 min. Another 2.5 mL of chloroform was added to the mixture and sonicated for 10 min. Then the mixture was centrifuged at 4000*g* at 4 °C for 5 min. Then the lower layer of 5 mL chloroform with extracted lipids were pipetted out for lipid analysis. The remaining cell pellets were re-extracted repeating the procedure.

### Method D: Dic/Met [34]

This method was the same as the Folch et al. method. However, all extractions used dichloromethane/methanol (2:1, v/v) instead of chloroform/methanol. In order to layering the extracted mixture, 1.625 mL KCL solution (0.88%) was used instead of 1.875 mL H_2_O. Lipids were then within the 5 mL dichloromethane phase. The remaining cell pellets were re-extracted repeating the procedure.

### Modified lipid extraction methods

Lipid extraction in the four test groups was carried out according to the four control groups (see above) with the following modifications. The 35 mg of dry microalgae samples were extracted two times as in the above-mentioned methods, but prior to the second solvent extractions, the pre-extracted fresh cell pellets were re-suspended in 5 mL dH_2_O (deionized) and vortexed for 30 s at room temperature, and then centrifuged at 4000*g* for 5 min at room temperature; the treatment was done only once. After centrifugation, the aqueous phase extractions were also kept for total lipids quantification, but the concentration levels were all around or below the detection limit, thus confirming that no detectable amounts of lipids were released in the water phase. Solvent phases obtained from the first and second extractions are defined as stage 1 and stage 2 respectively in both control and test groups.

### Lipid analysis

#### Fast total lipid assay

0.1 mL of extracted solvents were pipetted out from each solvent phase and evaporated under a stream of N_2_. Then each sample was re-suspended in 0.1 mL of acetone, and 0.9 mL of 1.5% sulfosalicylic solution was added. Each sample was shaken vigorously followed by a 30 min standing. The sample absorbance is read at 440 nm by UV–VIS determination (UNICAM 8625, UV/VIS) [35, 43], and then the quantification of the lipids is calculated according to a calibration curve (lipid concentration vs. absorption reading) using lipid extracted from *C. protothecoides* cells harvested at growth steady state [43]. For generating the calibration curve, known weighted lipids were dissolved in acetone to prepare a stock solution (2 g/L) and diluted to a series of standard solutions. The lipid concentration versus absorption reading was taken as a standard curve. Lipid quantification was thus done using this standard curve.

### Lipid class analysis

All remaining solvent phases (~4.9 mL) collected in each group were evaporated under a stream of N_2_ and each sample was re-suspended in 500 µL dichloromethane to analyze lipid classes. Lipid classes were identified by TLC–FID according to Parrish’s method [45].

### Fatty acids profiles analysis

Lipids were separated into polar (structural lipids, mainly phospholipids) and neutral fractions (including wax esters, sterols, free fatty acids and triglycerides) by column chromatography on silica gel micro-columns (30 × 5 mm I.D. Kieselgel 70–230 mesh Merck) as described in Marty’s method [46]. The neutral fraction was purified on an activated silica gel with 1 mL of hexane/ethyl acetate (v/v) to eliminate free sterols. FA composition of the neutral and the polar fractions were determined separately on fatty acid methyl esters (FAMEs) obtained by esterification using sulfuric acid/methanol (2:98, v/v), and then analyzed by GC–MS (Thermo Fisher Scientific Inc., GC model Trace GC Ultra and MS model ITQ900) [43, 47]. Standards for 37 fatty acids were used and only 32 fatty acids were detected in this work, listed as: C11:0_Undecanoic, C12:0_Lauric, C13:0_Tridecanoic, C14:0_Myristic, C14:1_Myristoleic, C15:0_Pentadecanoic, C15:1_cis-10-pentadecanoic, C16:0_Palmitic, C16:1_Palmitoleic, C17:0_Heptadecanoic, C17:1_Cis-10-heptadecenoic, C18:0_Stearic, C18:1n9_Oleic(c) + Elaidic(t), C18:2n6_Linolelaidic(t) + Linoleic(c), C18:3n6_Gamma-linolenic, C18:4n3_semi-quant, C19:0, C18:3n3_Alpha-Linolenic, C20:0_Arachidic, C20:1n9_Cis-11-eicosenoic, C20:2_Cis-11,14-eicosadienoic, C20:3n6_Cis-8,11,14-eicosatrienoic, C21:0_Henicosanoic, C20:4n6_Arachidonic, C20:3n3_Cis-11,14,17-eicosatrienoic, C20:5n3_cis-5,8,11,14,17-eicosapentaenoic, C22:0_Behenic, C22:1n9_Erucic, C22:2_Cis-13,16-docosadienoic, C24:0_Lignoceric, C22:6n_Cis-4,7,10,13,16,19-docosahexaenoic, C24:1n9_Nervonic.

### Statistical analysis

Three replicates were carried out for each experiment samples, and the variation within the replicates were assessed by calculating the standard deviation of the means. Evaluation of differences between the different extraction systems were carried out by analyses of variance (ANOVA) [34].

## Results

### H_2_O treatment significantly improves total lipid extraction yield

**Fig. 1:**
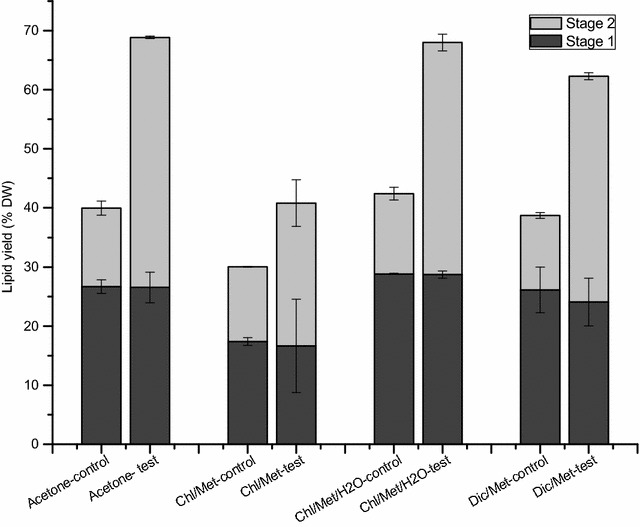
Total lipids extracted in stage 1 (*black*) and stage 2 (*grey*) for acetone method, Chl/Met method, Chl/Met/H_2_O method and Dic/Met method respectively, without (control) or with a water treatment (test)

In the present study, we evaluated a modification to current extraction methods for lipids in microalgae, adding a water treatment between two successive solvent extraction stages. The first solvent extraction stage was performed under the same condition in both control and test groups for the four different methods, with total lipids of 26.7 ± 1.1% DW in control and 26.5 ± 2.6% DW in test for method A; 17.4 ± 0.6% DW in control and 16.7 ± 7.9% DW in test for method B; 28.8 ± 0.1% DW in control and 28.7 ± 0.6% DW in test for method C; 26.1 ± 3.9% DW in control and 24.1 ± 4.0% DW in test for method D (Fig. 1). With the water treatment, test groups reached significantly higher total lipid levels compared to control, after the second solvent extraction stage. The total lipids yield in test group (42.3 ± 0.2% DW) was 3.2-fold that in control group (13.3 ± 1.2% DW) using acetone, 1.9-fold using Chl/Met (24.1 ± 4.0% DW in test and 12.6 ± 0.1% DW in control), 2.9-fold using Chl/Met/H_2_O (39.3 ± 13.5% DW in test and 13.6 ± 1.1% DW in control) and 3.0-fold using Dic/Met (38.2 ± 0.6% DW in test and 12.6 ± 0.5% DW in control). Lipid extraction efficiency thus improved by 72.3, 35.8, 60.3 and 60.9% respectively for acetone, Chl/Met, Chl/Met/H_2_O and Dic/Met by adding a water treatment between the two solvent extraction stages, which usually performed successively.

Attempts have been done to enhance lipid extraction yield by adding more solvent to wash the post-extracted biomass, or washing the post-extracted biomass with the extracted mixture (solvent and lipids mixture), but without any improvement [3]. Our results also show that in the control group, most of the extraction occurred in the first extraction step, with the second extraction yield only accounting for 31.2 ± 2.9% (13.3 ± 1.2% DW), 42.1 ± 1.1% (12.6 ± 0.1% DW), 32.0 ± 1.8% (13.6 ± 1.1% DW) and 32.5 ± 2.5% (12.6 ± 0.5% DW) of total extraction yield for acetone, Chl/Met, Chl/Met/H_2_O and Dic/Met methods respectively. However, in the test groups the second extraction stage following the water treatment accounted for 61.4 ± 2.4% (acetone), 59.2 ± 15.5% (Chl/Met), 57.7 ± 0.4% (Chl/Met/H_2_O) and 61.3 ± 3.6% (Dic/Met) of the final lipids yield.

Our data show that the total lipid extraction yield differs among the four original extraction methods. Lipid content in *C. protothecoides* biomass may rely on culture condition but it was reported reaching between 14.6 and 57.8 (%, w/wDW) [48], a range that is comparable with our data, in control groups. The yield obtained using the Chl/Met was significantly lower than those from Dic/Met (F_(1, 4)_ = 7.89, P < 0.05) and Chl/Met/H_2_O (F_(1, 4)_ = 249.93, P < 0.0001), which is in agreement with literature [3]. The extraction yield using acetone was also significantly higher than that from Chl/Met (F_(1, 4)_ = 639.15, P < 0.0001), but not statistically different to that from Chl/Met/H_2_O and Dic/Met method (F_(2, 6)_ = 1.08, P = 0.397). We then moved further characterizing the effect of the water treatment on extracted lipids composition.

### Water treatment promotes TAG-to-total lipid ratio in extraction processes

**Fig. 2:**
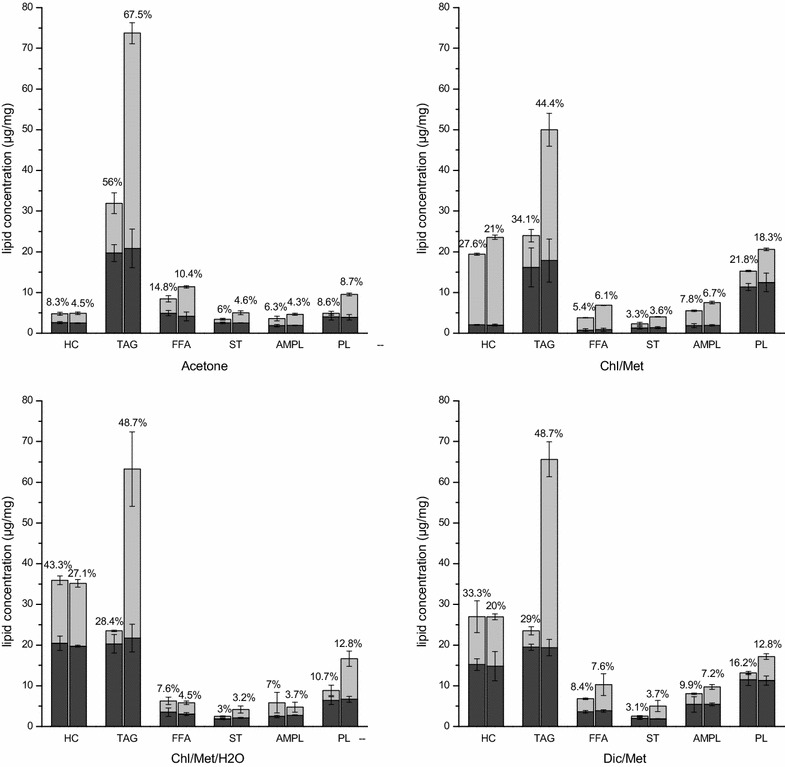
Lipids composition extracted in control (*left columns*) and test groups (*right columns*) for acetone method, Chl/Met method, Chl/Met/H_2_O method and Dic/Met method in the first (*black*) and second (*grey*) stage. The percentage represent extraction ratio for each lipid component in control and test group respectively

The major lipid classes identified include HC (hydrocarbons), TAG (triacylglycerols), FFA (free fatty acids), ST (sterols), AMPL (acetone mobile polar lipids) and PL (phospholipids) (Fig. 2). HC are mainly integrated in the cell membrane through amino acid residues anchored on it [49], TAG and ST are storage lipids, FFA are precursors of lipid synthesis, PL are the main component of cell membranes, whereas AMPL is a group constituted from glycolipids monoacylglycerols, pigments and degradation products of PLs [50]. Interestingly, in the first stage TAG was the main component extracted over total lipids, reaching a similar level of 19.4 ± 0.6 µg/mg in all four methods. However, the TAG content in total lipids extracted varied among the four methods with 55.3 ± 2.6% (acetone), 48.3 ± 5.7% (Chl/Met), 36.9 ± 0.1% (Chl/Met/H_2_O) and 34.0 ± 2.4% (Dic/Met). Moreover, HC was higher in Chl/Met/H_2_O (36.2 ± 1.0%) and Dic/Met (26.4 ± 0.2%), while PL was higher in Chl/Met (34.1 ± 0.2%) and Dic/Met (20.0 ± 0.1%). The water treatment affected differently the resulting lipid class distribution profile in the second solvent extraction phase depending on the method, but shows generally increased extraction yields. The second extraction stage led to significantly increased levels of HC in Chl/Met for both control (2.1 ± 0.1 µg/mg in stage 1 and 17.3 ± 0.3 µg/mg in stage 2) and test group (2.0 ± 0.2 µg/mg in stage 1 and 21.6 ± 0.5 µg/mg in stage 2). Using Chl/Met/H_2_O and Dic/Met also showed a high extraction efficiency for HC at the second stage with no significant effect of the water treatment, while acetone seems less efficient for HC extraction. Meanwhile, extraction of FFA, ST and AMPL was higher (or comparable) in the second stage for both control and test groups in all four methods. However, comparing control and test groups, HC extraction was only slightly improved in Chl/Met by water treatment (17.3 ± 0.3 µg/mg in control and 21.6 ± 0.5 µg/mg in test respectively), not significantly improved in acetone (2.2 ± 0.1 µg/mg in control and 2.4 ± 0.3 µg/mg in test respectively) and Dic/Met (11.8 ± 3.9 µg/mg in control and 12.1 ± 0.7 µg/mg in test respectively), while it was similar for Chl/Met/H_2_O (15.4 ± 1.1 µg/mg in control and 15.4 ± 0.9 µg/mg in test respectively). However, TAG, ST and PL revealed a high sensitivity to water treatment as showed by the significant extraction improvement in test groups compared to control groups in all four methods (Fig. 2). As the main component, TAG extraction was significantly improved compared to the other components (Table 1), with TAG levels of 4.3 ± 0.7 (acetone), 4.1 ± 0.3 (Chl/Met), 13.0 ± 3.5 (Chl/Met/H_2_O) and 11.5 ± 1.9-fold (Dic/Met) for the control groups in stage 2. Our results thus clearly show that the water treatment specifically favored the extraction of intracellular fractions of TAG, ST, and FFA compared to the membrane fractions of AMPL and HC (Table 1). Meanwhile, although PL, the main known cell membrane lipid component, reached 4.0 ± 1.9-fold the level in the control group, its extraction improvement was less than for TAG with an average of 8.2 ± 1.5-fold that in control group.

**Table 1:** Comparative extraction level as test-to-control (T/C) ratio for different lipid classes in stage 2

	TAG	FFA	ST	AMPL	PL	HC
Acetone	4.3 ± 0.7	2.1 ± 0.6	2.8 ± 0.2	1.6 ± 0.7	6.3 ± 4.5	1.1 ± 0.4
Chl/Met	4.1 ± 0.3	2.0 ± 0.0	2.7 ± 1.1	1.5 ± 0.0	2.1 ± 0.1	1.3 ± 0.1
Chl/Met/H2O	13.0 ± 3.5	1.0 ± 0.2	3.3 ± 0.9	0.6 ± 0.2	4.0 ± 0.2	0.8 ± 0.2
Dic/Met	11.5 ± 1.9	2.0 ± 0.9	5.8 ± 1.0	1.7 ± 0.3	3.6 ± 1.4	1.0 ± 0.5
Average	8.2 ± 1.5	1.8 ± 0.4	3.7 ± 0.8	1.3 ± 0.3	4.0 ± 1.9	1.1 ± 0.3

Results are expressed as the mean ± SD (n = 3)

Overall, combining the two extraction stages, the water treatment resulted in significantly higher TAG-to-total lipids ratios (67.5 ± 0.7%, 44.4 ± 3.9%, 48.7 ± 3.7% and 48.7 ± 0.1% for acetone, Chl/Met, Chl/Met/H_2_O and Dic/Met method respectively) compared to control (56.0 ± 5.0%, 34.1 ± 5.3%, 28.4 ± 2.3% and 29.0 ± 3.8% for acetone, Chl/Met, Chl/Met/H_2_O and Dic/Met method respectively), with reduction of HC-to-total lipids ratio of 3.8, 6.6, 16.2 and 13.3% for acetone, Chl/Met, Chl/Met/H_2_O and Dic/Met method respectively (Fig. 2). Of interest, acetone method with a water treatment resulted in the highest TAG extraction level with 73.7 ± 7.3 µg/mg, which is 130.8 ± 10.6% higher than the maximum value observed in all control groups (31.9 ± 4.6 µg/mg in acetone method).

Interestingly, when compared in parallel, our results confirm that each extraction method is specific to a lipid class (Fig. 2). For instance, the highest TAG extraction efficiency is for acetone method, reaching 56.0 ± 5.0% and 67.5 ± 0.7% in control and test group respectively, while it only reached 28.4 ± 0.7% in control and 48.7 ± 2.7% in test for Chl/Met/H_2_O. Acetone showed favoring extraction of ST and FFA, while not PL and HC (8.6 ± 1.7% and 8.3 ± 1.5% respectively in control, 8.7 ± 0.2% and 4.5 ± 0.2% respectively in test). Chl/Met method led to the highest extraction levels of PL and HC (21.8 ± 0.9% and 27.6 ± 3.6% respectively in control group, 18.3 ± 0.2% and 21.0 ± 1.8% respectively in test group). However, AMPL extraction level was similar in the four methods (Fig. 2). Results suggest that the different solvent and extraction procedures studied here have different selectivity for lipid components. Acetone may penetrate deeply and reach intracellular lipids, while Chl/Met and Dic/Met action may be mostly limited to membrane lipids.

### H_2_O treatment significantly favors neutral-to-polar lipid ratio extraction

Since fatty acids (FA) composition and structure, such as carbon chain length and unsaturated degree, greatly affect the properties of resulting biodiesel [51, 52], the FA profile was characterized for neutral (NL) and polar lipids (PL) independently (neutral and polar fractions were first separated as described in “Methods” section). A total of 32 FA were detected from C11 to C24 (as shown in “Methods” section), and similar fatty acids were found in NL and PL fractions with the five most prevalent components being C16:0, C18:0, C18:1n9, C18:2n6 and C18:3n3 in both NL and PL fractions. FAs are known as precursors of both neutral lipids and polar lipids, with no evidence of FAs selection priority during neutral lipid and polar lipid synthesis. Therefore, it was expected that similar FA components were found in both NL and PL.

**Fig. 3:**
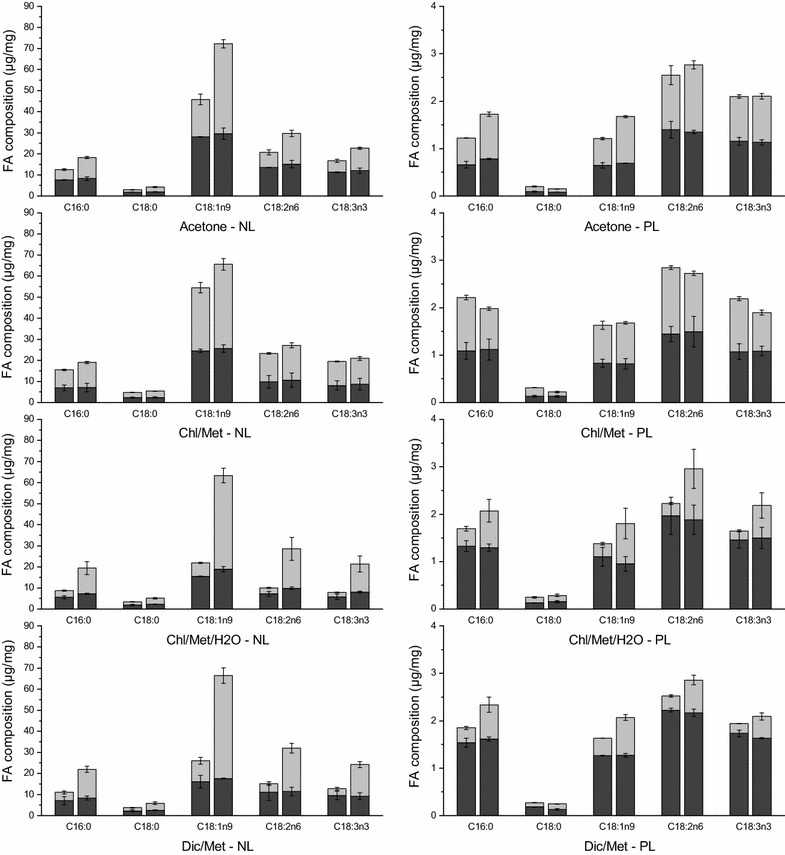
Five main fatty acids composition in neutral lipids fraction and polar lipids fraction respectively (control groups: *left columns*; test groups: *right columns*; first stage: *black*; second stage: *grey*)

The same result has also been reported in [24], where the most abundant FAs in the lipid extracts accounted for approx. 70% of total FAs, with C16 hexadecanoic acid, C18:1 (n-9) oleic acid and C18:2 (n-6) octadecadienoic acid. Interestingly, similar components of these dominant FAs were found in the four methods tested here. However, although the FAs in both fractions are quite similar, the quantity of each component differed in NL and PL fraction as shown in Fig. 3. For instance, the multi-unsaturated fatty acids C18:2n6 and C18:3n3 are clearly more abundant in PL than in NL, which suggests membrane lipids mobility. C16:0 is also more abundant in PL, which is maybe due to the fact that it is the initial FA synthesized and is first used for cell growth as in the structure of cell membrane.

C18:1n9 accounts for the highest content in the NL fraction, followed by C18:2n6 > C18:3n3 > C16:0 > C18:0, and this in all methods (Fig. 3). A water treatment resulted in a significant enhancement, at stage 2, of C18:1n9 in Chl/Met/H_2_O and Dic/Met methods (6.9 ± 1.5 and 4.9 ± 0.5-fold of that in control respectively), followed by acetone (2.4 ± 0.2-fold) and to a lesser extent in Chl/Met method (1.3 ± 0.2-fold). However, C18:1n9 reached a similar final extraction yield of 66.9 ± 1.9 µg/mg in all methods after water treatment. Indeed, C18:2n6, C18:3n3, C16:0 and C18:0 all showed similar trends with a significant improvement using Chl/Met/H_2_O and Dic/Met, than acetone and Chl/Met. However, fatty acids in PL fraction differ from that in NL fraction, with C18:2n6 the predominant component in all methods. With a water treatment, extraction efficiency of all five components were improved in acetone, Chl/Met/H_2_O and Dic/Met methods at different extents. However, Chl/Met method resulted in a slightly but significant lower extraction efficiency than the control group (Fig. 3). Adding a water treatment in Chl/Met method is thus detrimental to polar lipids extraction.

**Fig. 4:**
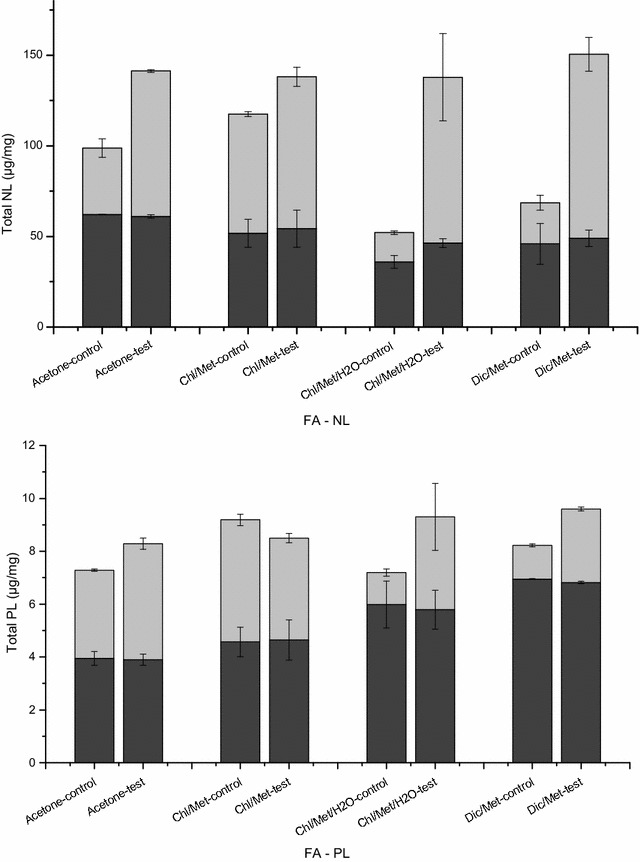
Sum of the fatty acids of neutral lipids fraction (FA-NL) and polar lipids fraction (FA-PL) in different methods

We then compared extraction methods analyzing the partition of extracted fatty acids in neutral lipids fraction (FA-NL) and in polar lipids fraction (FA-PL) (Fig. 4). Before H_2_O treatment, averaging the results in control and test samples, acetone method led to FA-NL extraction of 61.6 ± 0.6 µg/mg and FA-PL of 3.9 ± 0.1 µg/mg, corresponding to NL-to-PL ratio of 15.7 ± 0.1. However, in Chl/Met, Chl/Met/H_2_O and Dic/Met methods, NL-to-PL ratio is of 11.5 ± 0.2, 7.0 ± 1.0 and 6.9 ± 0.3 respectively, with less NL extracted (53.0 ± 1.8 µg/mg, 41.2 ± 7.3 µg/mg and 47.5 ± 2.2 ug/mg for Chl/Met, Chl/Met/H_2_O and Dic/Met methods respectively) but more PL extracted (4.6 ± 0.1 µg/mg, 5.9 ± 0.1 µg/mg and 6.9 ± 0.1 µg/mg for Chl/Met, Chl/Met/H_2_O and Dic/Met methods respectively). Acetone method thus shows the highest selectivity level for neutral lipids, with extraction yield ranked as acetone method > Chl/Met method > Dic/Met method > Chl/Met/H_2_O method. However, the PL extraction yield in stage one was ranked as Dic/Met method > Chl/Met/H_2_O method > Chl/Met method > acetone method (Fig. 4). For the second stage, results revealed that NL extracted in test groups (80.3 ± 0.7 µg/mg for acetone method, 83.8 ± 5.2 µg/mg for Chl/Met method, 91.5 ± 24.1 µg/mg for Chl/Met/H_2_O method and 101.5 ± 9.4 µg/mg for Dic/Met method) were increased compared to that in control groups (36.6 ± 5.1 µg/mg for acetone method, 65.7 ± 1.4 µg/mg for Chl/Met method, 16.1 ± 1.0 µg/mg for Chl/Met/H_2_O method and 22.7 ± 4.1 µg/mg for Dic/Met method). Indeed, a water treatment led to 2.1, 1.3, 5.1 and 3.8-fold that in control groups for acetone, Chl/Met, Chl/Met/H_2_O and Dic/Met method respectively. However, PL extraction in test groups was only improved in acetone, Chl/Met/H_2_O and Dic/Met methods (5.0 ± 0.5, 2.9 ± 0.7 and 2.2 ± 0.2-fold of control group for acetone, Chl/Met/H_2_O and Dic/Met methods respectively), and resulted in lower yields than control in Chl/Met method (0.8 ± 0.0 of that in control). Therefore, the NL-to-PL ratio is greatly improved with a water treatment (18.3 ± 1.0 for acetone method, 21.8 ± 0.6 for Chl/Met method, 26.0 ± 4.1 for Chl/Met/H_2_O method and 36.4 ± 6.1 for Dic/Met method) compared with control (11.0 ± 2.6 for acetone method, 14.2 ± 1.4 for Chl/Met method, 13.3 ± 1.0 for Chl/Met/H_2_O method, 17.8 ± 3.4 for Dic/Met method). Of interest, the neutral lipids fraction is mainly stored in the cell while polar lipids fraction is mainly within the cell membrane, suggesting H_2_O treatment favors the release of intracellular storage lipids.

## Discussion

The key step in the extraction and recovery of lipids from microalgae relies on their release from intracellular compartment, where stands the major lipid pool [3]. Moreover, the extraction process efficiency, which is also a mass transfer operation problem, largely depends on the nature of the solvent as shown in this work as well as in the cited literature. Therefore, each method is expected to display a specific selectivity for each compound to extract. In this work on *C. protothecoides*, lipid extraction yields efficiency is ranked as acetone-based method > Chl/Met/H2O method > Dic/Met method > Chl/Met method. This ranking agrees with the polarity degree of the extraction solvents; acetone and Chl/Met/H_2_O polarity being higher than Chl/Met and Dic/Met. Indeed, our results suggest that the water treatment increases solvent mixtures polarity and thus explains the resulting enhanced extraction yields. Moreover, since the cell membrane mainly contains polar lipids, the use of polar solvents could increase lipids diffusion phenomenon, as suggested by Araujo for acetone [3]. It has been already observed that nonpolar solvents have lower selectivity levels toward microalgae lipids compared to polar solvents [3]. This relationship has also been suggested in other reports. Li [24] observed that an hexane and ethanol mixture resulted in two times higher lipid yields than hexane in *Tetraselmis sp.*, a result that the authors explained by the lower polarity of hexane over the hexane & ethanol mixture. Rychecosch et al. [39] and Lewis et al. [28] also demonstrated that a mixture of polar and non-polar solvents succeeded at extracting higher amounts of lipids compared to non-polar solvents. However, contradictory results have also been reported but for other microalgae species. For instance, Shen et al. [53] showed that an hexane and ethanol mixture extracted less lipid than hexane on *C. protothecoides* and *Scenedesmus dimorphus*. Structural and composition differences of algal species may explain differences in extraction protocols efficiencies.

Pure H_2_O is a polar solvent having a high activity level that is thought to contribute perturbing cell membrane permeability, which is already highly weakened from the use of solvents in stage one. In addition, a hypotonic environment generated adding pure water results in the increase of cell volume (Additional file 1: Figure S1) to equilibrate osmotic pressure, a phenomenon which greatly affects membrane integrity. Solvents access to the cell interior volume is then made easier. The combination of stressful phenomena may thus explain improving total lipid yield with the water treatment. In addition, re-suspending the cells in pure H_2_O may increase the polarity of the cellular microenvironment in the second stage extraction, which further favors the lipid diffusion process out of the cell volume. All of the above can thus explain that extraction of intracellular TAG, ST and FFA are preferentially increased compared to membrane lipids such as HC, PL and AMPL after water treatment.

It is thus clear from this work (Fig. 2) as well as from literature that each extraction protocol may differ in its selectivity for the different lipid classes found in microalgae. HC is a non-polar component anchored on the cell membrane by amino acids residues, and should then be more available to the less polar solvent mixtures Chl/Met and Dic/Met. However, although this is the case for Dic/Met method, results for Chl/Met and Chl/Met/H_2_O revealed Chl/Met is quite selective for HC when residual water remains with the cell pellets. This may be due to the fact that the non-polar HC is embedded in the polar phospholipids layers by amino acids residues. The presence of water may thus increase solvent mixture polarity and help weakening the links between polar lipids and proteins anchored into the membrane, hence making HC (neutral) more available to the less-polar solvent mixture Chl/Met. However, two times successive solvent extraction stages shown leading to a similar effect, as shown in Chl/Met and Chl/Met/H_2_O with the release of HC from the membrane, no matter whether water treatment is applied or not.

Finally, although H_2_O treatment could lead to different lipid class compositions and significantly improve the sum of fatty acids extracted, the effect on the FA composition was less important. The most abundant FAs in the lipid extracts include C16:0, C18:1n9, C18:2n6 and C18:3n3. The FAs composition was not affected by the water treatment, with final FAs composition in each method being similar in control and test groups. For instance, acetone method led to 12.7% of C16:0 in control group and 12.4% in test group, 46.4% of C18:1n9 in control group and 49.1% in test group, 21.0% of C18:2n6 in control group and 20.2% in test group, 16.9% of C18:3n3 in control group and 15.4% in test group, and ~ 3.0% of other fatty acids in both control and test groups. Moreover, FAs composition was also found similar in the four methods, modified or not, compared stage by stage, which suggests that different extraction methods studied have limited impact on FAs composition selectivity, as proposed by Li [24]. The most abundant FAs extracted in the four methods are fortunately the ones preferred for microalgae biodiesel production [54].

**Fig. 5:**
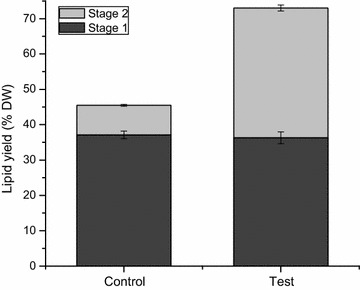
Total lipids extracted in stage 1 (*black*) and stage 2 (*grey*) using fresh biomass for acetone method, without (control) or with a water treatment (test)

In the present work, we have clearly demonstrated that the classical extraction methods can be significantly improved from the addition of a water treatment between the two solvent extraction steps. However, all these methods were historically based on the use of dry microalgae biomass, while recent developments in the field propose the use of fresh biomass. Avoiding the drying process allows reducing process energy and costs, as well as it enables a positive energy balance between the process energy and that extracted from the microalgae biomass (e.g. biodiesel) [55]. Therefore, in complement to assessing classical methods which are based on using dry biomass, we have evaluated the effect of adding a water treatment using fresh biomass on a modified acetone-based extraction method, and obtained 1.6-fold total lipid extracted with water treatment (Fig. 5). Indeed, in addition to significantly improving the lipid extraction efficiency, with over 100% increase of the harvested TAG, a precursor leading to biodiesel, the addition of a water treatment step is thus expected to enhance significantly the global final energy yield (e.g. of ~100% estimated from the experimental results in this work) also while avoiding energy consumption for drying the algal cells before the solvents extraction steps. To conclude, the global process may then turn out to be positive energetically speaking, and the energy cost should be greatly lower than for the classical methods. Except for energy, the other part of costs difference between the new protocol proposed here and classical methods rely on equipment investment, from biomass pre-treatment to the extraction process. Adding a water treatment step will specifically require a water deionisation system, which would most likely be already available for other uses in the biological production plant, but will not need a cell dryer equipment such as in classical methods. Therefore, the equipment investment is similar when adding a water treatment step.

Finally, recent approaches propose replacing the use of ultrasounds to perform microalgae cells disruption [56, 57] with “green solvents” such as 1-butyl-3-methylimidazolium chloride [56–58]. These solvents are capable of lysing microalgae cell walls and microalgae vesicle membranes and thus favor the release of the cell lipids [56]. In fine, it is believed that the addition of a water treatment can allow to enhance lipid extraction efficiency, and thus improve the productivity of a biodiesel production process based on microalgae biomass.

## Conclusion

Through the modification of four classical lipids extraction methods this study clearly demonstrated that water treatment of biomass after the first solvent extraction phase favors the release of intracellular lipids in the second solvent extraction step. Total lipid extraction yield as well as intracellular lipid class ratios in the final extract were thus significantly increased by the water treatment. The neutral-to-polar lipid ratio is also greatly improved after the water treatment, and the preferable lipid component TAG showed being increased up to 130.8% compared to the original extraction methods. H_2_O treatment between two-stage solvent extraction processes thus allows increasing the extraction efficiency, most probably through further perturbing cell membrane porosity and integrity. Furthermore, re-suspending the cells in pure H_2_O before extraction stage 2 increases the polarity of solvent mixture in the cellular vicinity thus enhancing the second stage extraction efficiency. The selection of the proper solvent system is crucial to the extraction process, because it may affect solvent penetration of the cell membrane and therefore lipids extraction. Finally, we conclude inviting to re-visit current productivity levels of microalgae bioprocesses by modifying extraction protocols adding a water treatment.

## Additional file

**Additional file 1.** Microscopic images of cells.doc. Figure S1. Cells before (a) and after (b) H_2_O treatment step, 400X magnification under bright field optical microscopy (Leitz Laborlux S Microscope).

## Abbreviations

Chl/Met: chloroform/methanol
Chl/Met/H_2_O: chloroform/methanol/H_2_O
Dic/Met: dichloromethane/methanol
HC: hydrocarbons
TAG: triacylglycerols
FFA: free fatty acids
ST: sterols
AMPL: acetone mobile polar lipids
PL: phospholipids
FAMEs: fatty acid methyl esters
