# Supplementary material for: Current lipid extraction methods are significantly enhanced adding a water treatment step in Chlorella protothecoides
Source: Microb Cell Fact. 2017 Feb 11;16:26. doi: 10.1186/s12934-017-0633-9 (PMC5303247; doi:10.1186/s12934-017-0633-9)

Figure S1. Cells before (a) and after (b) H_2_O treatment step, 400X magnification under bright field optical microscopy (Leitz Laborlux S Microscope).


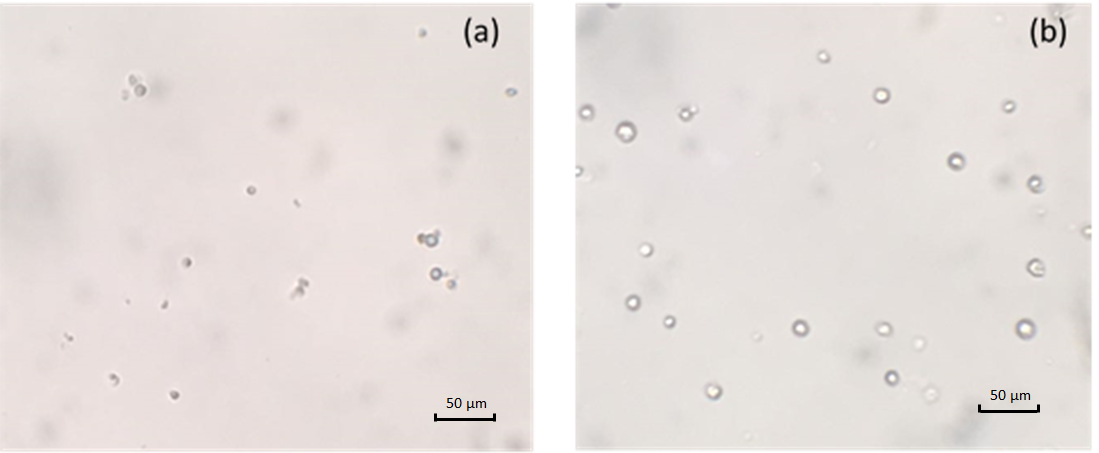

Supplement: Supplementary file 1 — Additional file 1. Microscopic images of cells.doc. Figure S1. Cells before (a) and after (b) H2O treatment step, 400X magnification under bright field optical microscopy (Leitz Laborlux S Microscope). [file 12934_2017_633_MOESM1_ESM.docx]
